# Functional validation of a novel *AAAS* variant in an atypical presentation of Allgrove syndrome

**DOI:** 10.1002/mgg3.1966

**Published:** 2022-05-15

**Authors:** Erica L. Macke, Joel A. Morales‐Rosado, Sarah K. Macklin‐Mantia, Christopher T. Schmitz, Björn Oskarsson, Eric W. Klee, Klaas J. Wierenga

**Affiliations:** ^1^ Center for Individualized Medicine Mayo Clinic Rochester Minnesota USA; ^2^ Department of Quantitative Health Sciences Mayo Clinic Rochester Minnesota USA; ^3^ Department of Medical Genetics Mayo Clinic Jacksonville Florida USA; ^4^ Department of Neurology Mayo Clinic Jacksonville Florida USA; ^5^ Department of Clinical Genomics Mayo Clinic Rochester Minnesota USA

**Keywords:** achalasia, alacrima, aladin, allgrove, whole‐exome sequencing

## Abstract

**Background:**

Achalasia‐addisonianism‐alacrima syndrome, frequently referred to as Allgrove syndrome or Triple A syndrome, is a multisystem disorder resulting from homozygous or compound heterozygous pathogenic variants in the gene encoding aladin (*AAAS*). Aladin is a member of the WD‐repeat family of proteins and is a component of the nuclear pore complex. It is thought to be involved in nuclear import and export of molecules. Here, we describe an individual with a paternally inherited truncating variant and a maternally inherited, novel missense variant in *AAAS* presenting with alacrima, achalasia, anejaculation, optic atrophy, muscle weakness, dysarthria, and autonomic dysfunction.

**Methods:**

Whole‐exome sequencing was performed in the proband, sister, and parents. Variants were confirmed by Sanger sequencing. The localization of aladin to the nuclear pore was assessed in cells expressing the patient variant.

**Results:**

Functional testing of the maternally inherited variant, p.(Arg270Pro), demonstrated decreased localization of aladin to the nuclear pore in cells expressing the variant, indicating a deleterious effect. Follow‐up testing in the proband's affected sister revealed that she also inherited the biallelic *AAAS* variants.

**Conclusions:**

Review of the patient's clinical, pathological, and genetic findings resulted in a diagnosis of Triple A syndrome.

## Graphical abstract

An individual presenting with alacrima and achalasia was found to harbor two variants *AAAS*. The missense variant falls into one of the WD‐repeat regions (bottom, purple). Functional assessment of the missense variant p.(Arg270Pro) indicates that this variant results in mislocalization of aladin (green), with mutant cells showing diffuse staining in the cytoplasm while in wild type cells aladin localizes to the nuclear pore (red). This case highlights the importance of careful consideration of variants in *AAAS* in the absence of all three classical features and demonstrates the utility of research‐based testing for the functional impact of variants of uncertain significance.

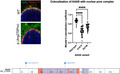

## 1 BACKGROUND

Achalasia‐addisonianism‐alacrima syndrome (MIM: 231550), frequently referred to as Allgrove syndrome or Triple A syndrome, is a multisystem disorder described by Allgrove et al. (1978), at which time the genetic etiology was unknown (Allgrove et al., 1978). Allgrove syndrome is typically characterized by the presence of three classical features—esophageal achalasia, alacrima, and adrenal insufficiency due to adrenocorticotropin hormone (ACTH) insensitivity (Allgrove et al., 1978; Handschug et al., 2001; Houlden et al., 2002). Allgrove syndrome has a wide and variable phenotypic spectrum, with patients presenting additional features including autonomic dysfunction, short stature, microcephaly, optic atrophy, dermatologic abnormalities, and muscle weakness and atrophy (Handschug et al., 2001; Houlden et al., 2002; Kimber et al., 2003; Milenkovic et al., 2010). Typically alacrima present in infancy, while achalasia and adrenal insufficiency may appear in childhood or adulthood (Gaiani et al., 2020; Kimber et al., 2003; Milenkovic et al., 2010). All three classical features are observed in approximately two thirds of cases, with the remainder presenting only two classical symptoms (Roucher‐Boulez et al., 2018).

Pathogenic homozygous or compound heterozygous variants in the gene encoding aladin (*AAAS*) have been shown to cause Allgrove Syndrome (Handschug et al., 2001; Tullio‐Pelet et al., 2000). Most pathogenic variants described are frameshift, nonsense, or splice site variants that result in a loss of gene function (Handschug et al., 2001; Houlden et al., 2002; Kurnaz et al., 2018; Milenkovic et al., 2010; Roucher‐Boulez et al., 2018; Sandrini et al., 2001; Singh et al., 2018; Tullio‐Pelet et al., 2000). Less commonly missense variants have been associated with Allgrove Syndrome (Houlden et al., 2002; Krumbholz et al., 2006; Roucher‐Boulez et al., 2018). There is no correlation between variant type or location and clinical phenotype, and individuals with the same variants may exhibit phenotypic variability (Goizet et al., 2002; Sandrini et al., 2001).

*AAAS* encodes Aladin WD Repeat Nucleoporin (aladin), which is a member of the WD‐repeat family of proteins. The aladin protein is highly conserved in eukaryotes and is a nucleoporin involved in the formation of the nuclear pore complex (NPC). It is thought to be involved in nucleocytoplasmic transport. The C‐terminal protein domain is necessary but not sufficient for localization to the NPC (Cronshaw & Matunis, 2003). The WD‐repeats have also been shown to be involved in NPC localization (Cronshaw & Matunis, 2003; Krumbholz et al., 2006). Pathogenic variants in *AAAS* reduce localization of aladin to the NPC, and increase aladin within the cytoplasm (Cronshaw & Matunis, 2003; Krumbholz et al., 2006). This is mediated by loss of function variants as well as missense variants within the WD‐repeats (Cronshaw & Matunis, 2003; Krumbholz et al., 2006).

## 2 MATERIALS AND METHODS

### 2.1 Ethical compliance

The proband and family members provided written consent following Mayo Clinic Institutional Review Board approval.

### 2.2 Whole‐exome sequencing analysis

Genomic DNA from the proband, parents, and affected sibling was extracted from blood. The DNA was enriched for the complete coding regions and splice site junctions for most genes of the human genome using a proprietary capture system developed by GeneDx for next‐generation sequencing with CNV calling (NGS‐CNV). The enriched targets were simultaneously sequenced with paired‐end reads on an Illumina platform. Bi‐directional sequence reads were assembled and aligned to reference sequences based on NCBI RefSeq transcripts and human genome build GRCh37/UCSC hg19. Data were filtered and analyzed to identify sequence variants. Reported clinically significant variants were confirmed by an appropriate orthogonal method in the proband and family members.

### 2.3 Generation of GFP‐tagged construct, immunocytochemistry, and quantification

Human AAAS (NM_015665.5) in pcDNA3.1(+)‐N‐eGFP constructs were purchased from Genscript to contain the wild‐type sequence, or with variants c.809G > C p.(Arg270Pro), or c.500C > T p.(Ala167Val).

HeLa cells were plated at 5 × 10^5^ cells per well on poly‐D‐lysine (Sigma, Cat No. P6407)‐coated glass coverslips and incubated at 37°C overnight. The following day, the cells were transfected with the GFP‐tagged constructs using Lipofectamine 3000 reagent (Invitrogen, Cat No. L3000008). After 48 hours, cells were fixed in 4% formaldehyde and permeabilized with 0.2% Triton X‐100. Cells were stained with nucleoporin p62 (Nup62) antibody diluted 1:400 (BD Biosciences, Cat No. 610497) at 4°C overnight. The slides were then incubated with goat anti‐mouse IgG Alexa Fluor 555 secondary antibody (Invitrogen, Cat No. A21424) diluted 1:500 for 1 hour at room temp. Slides were then mounted in Vectashield with DAPI (Vector Laboratories, Cat No. H‐1500) and images were obtained using an LSM 980 with Airyscan 2 confocal microscope with a 63×/oil objective (ZEISS). Quantification was measured by obtaining the Mander's co‐localization coefficient of Nup62 with aladin protein for individual cells of each construct AAAS wild‐type, p.A167V, and p.R270P (n = 42 cells, 48 cells, and 38 cells, respectively) using the ZEN imaging software (ZEISS) and analyzed in GraphPad Prism using ANOVA to compare the variants to wild‐type (*p < .05, **p < .01, ***p < .001. ****p < .0001).

## 3 RESULTS

### 3.1 Case presentation

The proband presented to the genetics clinic at age 29 to seek a diagnosis for a history of seizures, achalasia, optic atrophy, muscle weakness, neuropathy, and autonomic dysfunction. The proband was born at 36 weeks gestation and required a brief NICU stay due to hypotonia and poor feeding. There were no additional complications, and initial milestones in infancy were normal. He had lifelong hypolacrimation with a recent abnormal Schirmer tear test. At 11 months of age he had his first seizure, with many thereafter. The seizures were initially thought to be febrile in nature, but later when it became clear that he had a true seizure disorder, he was diagnosed with epilepsy. He was treated with phenobarbital until age eight, at which time treatment was discontinued due to lack of seizure activity. He has not had seizures since. At age 11 he developed achalasia and dysphagia. MRI of his chest showed a dilated esophagus in keeping with the reported achalasia, which was corrected with a Heller myotomy. Around age 11 visual problems emerged, and he was diagnosed with optic atrophy. Distal muscle weakness started at age 18 and has progressed over time. Around age 24 the weak distal muscles were atrophying, and dysarthria began. He also developed a tingling sensation in his hands. The proband has a longstanding history of erectile dysfunction and the inability to ejaculate. From a GI standpoint he has a longstanding history of constipation. We performed autonomic reflex screening at age 28 which showed a moderate autonomic neuropathy. Brain MRI showed a left frontal lobe developmental venous anomaly, which was assessed as incidental both in regard to his epilepsy and later his AAAS. His electromyography and nerve conduction studies at age 29 were abnormal and indicated a very chronic, slowly evolving, or static motor predominant neurogenic process. Currently, the proband continues to have GI discomfort, pain, and constipation. The intermittent tingling and numbness in his hands have increased in frequency. He has not developed any new medical problems since genetic testing was completed, and still does not have any identifiable endocrine abnormality.

### 3.2 Family history

**FIGURE 1:**
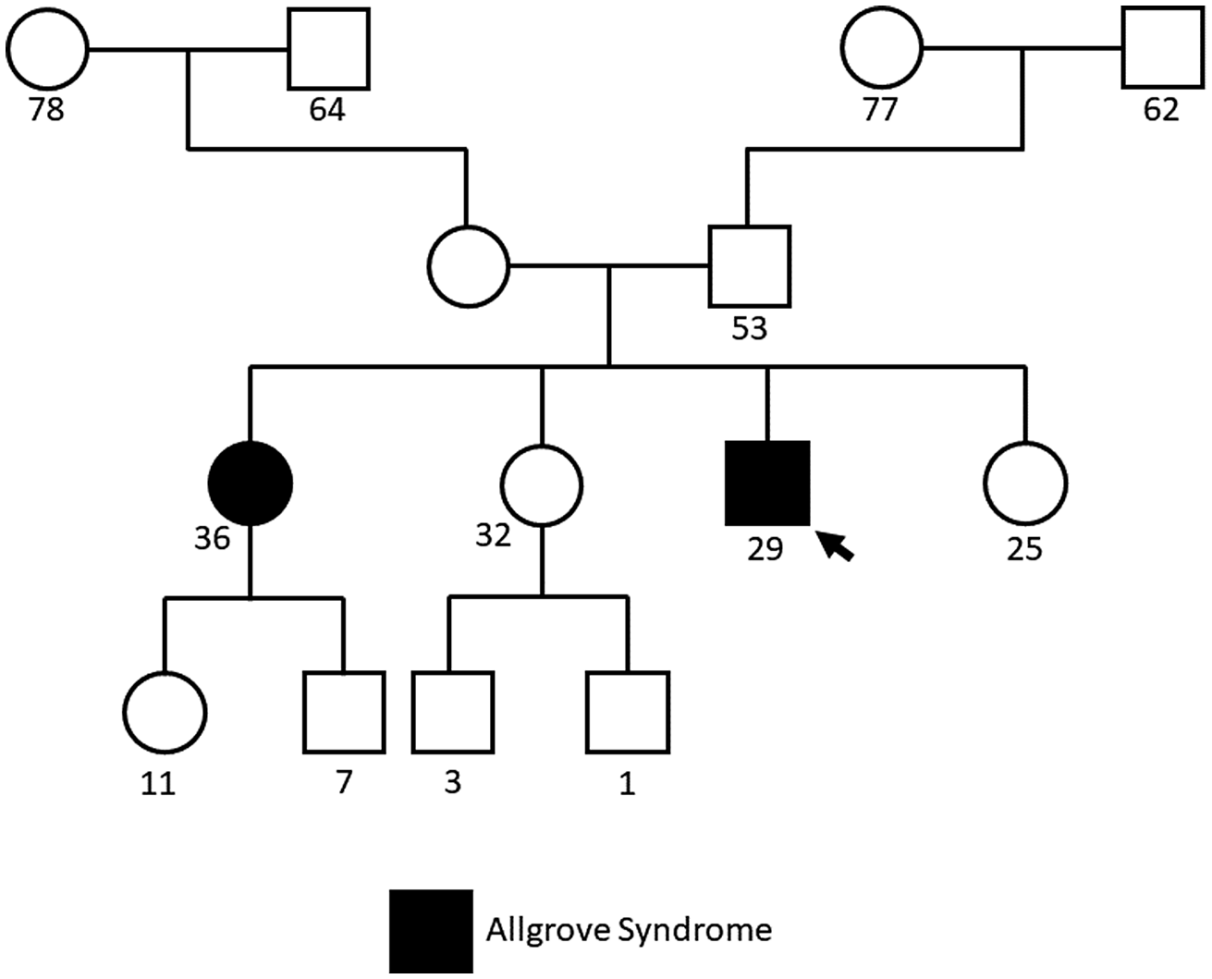
Family pedigree for the analyzed proband. The proband is indicated with a black arrow. Numbers underneath indicate years of age

The proband's parents did not present with any shared symptoms with the proband. He had three sisters, two of whom are healthy, and one who exhibits similar phenotypes to the proband, including alacrima, achalasia, and slurred speech (Figure 1). Overall, her presentation has been reported by family members to be milder than the proband.

### 3.3 Genetic analysis and functional assessment of a *AAAS* VUS

A pathogenic variant, c.211delC, p.(His71Ilefs*23) in *AAAS* (NM_015665.5), was inherited from the proband's unaffected father (Table 1). This variant had been previously observed in family with Allgrove syndrome (Houlden et al., 2002). A second *AAAS* variant, c.809G > C, p.(Arg270Pro), was inherited from the proband's unaffected mother (Table 1). This alteration was classified as a variant of uncertain significance (VUS). The proband's affected sister was also found to be compound heterozygous for these variants.

**TABLE 1:** Genomic findings

Gene	Transcript	HGVS cDNA	HGVS protein	Zygosity	Inheritance	ACMG classification
*AAAS*	NM_015665.5	c.211delC	p.His71Ilefs*23	Heterozygous	Paternal	Pathogenic
*AAAS*	NM_015665.5	c.809G > C	p.Arg270Pro	Heterozygous	Maternal	VUS

Abbreviations: ACMG, American College of Medical Genetics and Genomics; HGVS, Human genome variation society.

The maternally inherited VUS was not observed in large population cohorts (Lek et al., 2016), and in silico tools were conflicting in regards to its impact on protein structure and function. Conservation‐based tool SIFT and a high CADD Phred Score (23.6) suggested that the variant is deleterious. In contrast, Polyphen2, MutationTaster, and PredictSNP2 suggested that the variant is tolerated. The altered arginine residue is conserved in chimps and orangutans, but not across other mammalian orthologues. This residue falls within exon 8 of 16 total *AAAS* exons, and in one of the WD‐repeats. It is not predicted to impact splicing (Jaganathan et al., 2019).

**FIGURE 2:**
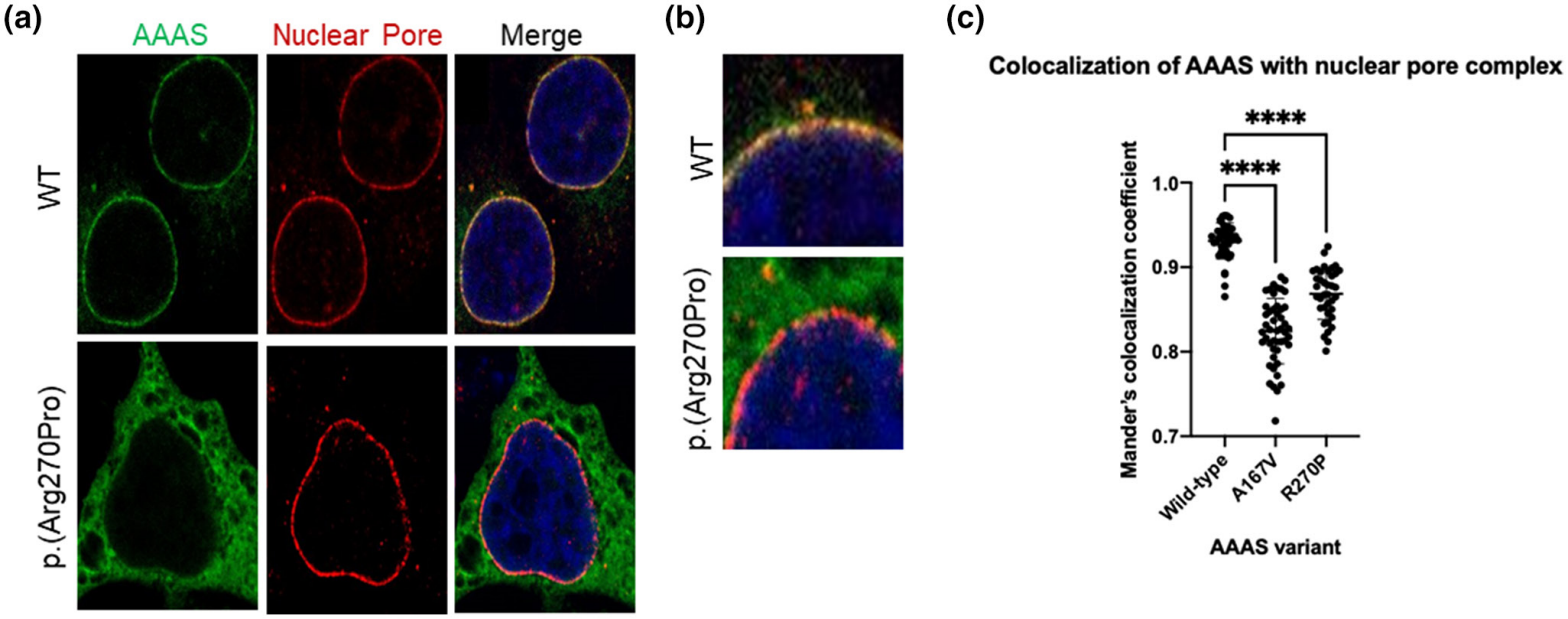
Functional assessment of *AAAS* VUS. (a) HeLa cells with GFP‐tagged wild‐type or mutant aladin (green) and NUP62 (red) labeling the NPC. (b) Insert of the merged panel from 2A showing co‐localization of aladin and NUP62 in wild‐type cells (yellow) and significantly reduced co‐localization in cells expressing the proband's variant. (c) Quantification of co‐localization of aladin and NUP62 in wild‐type cells, cells expressing a pathogenic variant (positive control), and cells expressing the patient variant

To further understand the functional impact of this VUS, a GFP‐tagged construct containing the p.(Arg270Pro) variant was expressed in HeLa cells, and aladin protein localization was observed. In control cells aladin co‐localizes with nucleoporin 62 (NUP62) at the nuclear pore (Figure 2). In positive control cells expressing a known pathogenic missense variant, p.(Ala167Val), and cells expressing the p.(Arg270Pro) variant, we observed a significant loss of co‐localization with NUP62 and mislocalization of aladin to the cytoplasm (Figure 2). These data indicate that this variant results in aberrant localization of the aladin protein.

## 4 DISCUSSION AND CONCLUSIONS

Here, we describe an individual presenting with two of the classical features of Allgrove syndrome, along with several of the less common phenotypes. WES was used to identify a novel missense variant in *AAAS*, p.(Arg270Pro). The variant is compound heterozygous with p.(His71Ilefs*23), previously observed in individuals with Allgrove syndrome. Functional assessment of the novel missense variant indicates that it disrupts the subcellular localization of aladin. While missense variants are less commonly associated with Allgrove syndrome, previously described missense variants have also been identified in the WD‐repeats (Krumbholz et al., 2006; Milenkovic et al., 2010; Tullio‐Pelet et al., 2000). WD‐repeat proteins are involved in a variety of cellular processes, and most form a propeller structure requiring a minimum of four WD‐repeats (Smith et al., 1999). It is possible that these variants result in disruption of the WD‐repeat propeller structure assembly.

The maternally inherited missense variant was initially classified as a VUS by a reference laboratory using the American College of Medical Genetics (ACMG) criteria of PM2, PM3, PP4, and BP4 (Richards et al., 2015). Functional assessment of this variant using previously established methods (Krumbholz et al., 2006) with inclusion of positive and negative controls allows for the application of PS3 supporting criteria (Brnich et al., 2019; Richards et al., 2015), elevating this variant to likely pathogenic. While the affected sister harbors both of the *AAAS* variants, PP1 criteria is not able to be applied due to insufficient numbers of affected family members to test for segregation.

Interestingly, the proband does not exhibit the adrenal dysfunction classically seen in Triple A syndrome. He has had several instances of adrenocorticotropic hormone testing, each of which was normal (Supplemental Table S1). However, it is important to note that the proband is relatively young, and although unlikely, adrenal insufficiency could still present later in life. There have been rare reports of Triple A patients presenting with only alacrima and achalasia, and only two of the classical criteria are required for diagnoses (Gaiani et al., 2020; Rivera‐Suazo et al., 2020). This highlights the importance of careful consideration of variants in AAAS in the absence of all three classical features and demonstrates the utility of research‐based testing for the functional impact of VUSs. While the patient only exhibits two of the classical features, his expanded phenotype is consistent with additional features of Allgrove syndrome reported in the literature. Specifically, his autonomic dysfunction, optic atrophy, anejaculation, and amyotrophy are all consistent with previously reported cases. Review of the patients clinical, pathological, and genetic findings resulted in a diagnosis of Allgrove syndrome for the proband.

## CONFLICT OF INTEREST

The authors declare that they have no competing interests.

## AUTHOR CONTRIBUTIONS

Erica L. Macke analyzed data and drafted the manuscript. Erica L. Macke, Christopher T. Schmitz, and Eric W. Klee contributed to data collection and analysis. Sarah K. Macklin‐Mantia, Björn Oskarsson, and Klaas J. Wierenga carried out family recruitment and clinical analysis. Eric W. Klee and Klaas J. Wierenga supervised the work. All authors read and approved the final manuscript.

## Supporting information


TABLE S1

## Data Availability

The data that support the findings of this study are available from the corresponding author upon reasonable request.
