# Supplementary material for: Functional validation of a novel AAAS variant in an atypical presentation of Allgrove syndrome
Source: Mol Genet Genomic Med. 2022 May 15;10(7):e1966. doi: 10.1002/mgg3.1966 (PMC9266593; doi:10.1002/mgg3.1966)
Supplement: Supplementary file 1 — TABLE S1 [file MGG3-10-e1966-s001.docx]

**Supplementary Information**

| **Age** | **28y** | **28y** | **29y** | **29y** |
| --- | --- | --- | --- | --- |
| **ACTH (pg/mL)** | 17 | 21 | 33 | 11 |

Supplementary Table S1: Adrenocorticotropic Hormone, reference range 7.2-63pg/mL.
